# α-Actinin-4 Enhances Colorectal Cancer Cell Invasion by Suppressing Focal Adhesion Maturation

**DOI:** 10.1371/journal.pone.0120616

**Published:** 2015-04-10

**Authors:** Miki Fukumoto, Shusaku Kurisu, Tesshi Yamada, Tadaomi Takenawa

**Affiliations:** 1 Integrated Center for Mass Spectrometry, Kobe University Graduate School of Medicine, 7-5-1 Kusunoki-cho, Chuo-ku, Kobe, Hyogo 650–0017, Japan; 2 Biosignal Research Center, Organization of Advanced Science and Technology, Kobe University, 1–1 Rokkodai-cho, Nada-ku, Kobe, Hyogo 657–8501, Japan; 3 Pathology Division, National Cancer Center Research Institute, Tokyo 104–0045, Japan; INRS, CANADA

## Abstract

α-Actinins (ACTNs) are known to crosslink actin filaments at focal adhesions in migrating cells. Among the four isoforms of mammalian ACTNs, ACTN1 and ACTN4 are ubiquitously expressed. Recently, ACTN4 was reported to enhance cancer cell motility, invasion, and metastasis. However, the mechanism by which ACTN4 drives these malignant phenotypes remains unclear. Here, we show that ACTN4, but not ACTN1, induces the formation of immature focal adhesions in DLD-1 cells, leading to the rapid turnover of focal adhesions. Interestingly, zyxin (ZYX) assembly to focal adhesions was markedly decreased in ACTN4-expressing DLD-1 cells, while the recruitment of paxillin (PAX) occurred normally. On the other hand, in ACTN1-expressing DLD-1 cells, PAX and ZYX were normally recruited to focal adhesions, suggesting that ACTN4 specifically impairs focal adhesion maturation by inhibiting the recruitment of ZYX to focal complexes. Using purified recombinant proteins, we found that ZYX binding to ACTN4 was defective under conditions where ZYX binding to ACTN1 was observed. Furthermore, Matrigel invasion of SW480 cells that express high endogenous levels of ACTN4 protein was inhibited by ectopic expression of ACTN1. Altogether, our results suggest that ZYX defective binding to ACTN4, which occupies focal adhesions instead of ACTN1, induces the formation of immature focal adhesions, resulting in the enhancement of cell motility and invasion.

## Introduction

α-Actinins (ACTNs) are ubiquitously expressed cytoskeleton proteins that crosslink actin filaments at adherence junctions in epithelial cells and focal adhesions in polarized migrating cells [1,2]. In focal adhesions, ACTNs interact with a variety of other focal adhesion-associated proteins such as vinculin (VCL) [3,4] and integrins [5,6], and then link actin filaments to focal adhesions [7–9]. There are four isoforms of ACTNs in mammalian cells [10–12]. ACTN1 and ACTN4 are ubiquitously expressed and are called non-muscle isoforms, while ACTN2 and ACTN3 are specifically expressed in muscle tissues. Among ACTNs, ACTN4 is primarily involved in cell motility and cancer invasion [12–21]. During cell movement, ACTN4 protein expression level is markedly increased and ACTN4 concentrates at the leading edge of migrating cells [12]. ACTN4 knockdown suppresses the migration and invasion of cancer cells [15–18,20–22], whereas its overexpression in colorectal cancer cells induces lymph node metastasis in immunodeficient mice [13]. Furthermore, ACTN4 protein expression is closely related to poor outcome in patients with breast [12], colorectal [13], pancreatic [20,23], ovarian [19], bladder [21], and lung [24] cancer. However, the reason why ACTN4, rather than ACTN1, is frequently associated with cancer malignancies despite similarities in domain structure, actin-binding and-crosslinking activities, and Ca^2+^-sensitivity between the two remains to be elucidated [25].

Focal adhesions are large integrin-based, dynamic macromolecular structures that connect the extracellular matrix with the intracellular bundles of actin filaments called stress fibers. Focal adhesion is the primary structure that transmits extracellular tensile force into a cell. Thus, the adhesive strength of cells to the substrate and the lifetime or dynamics of focal adhesions critically affects the dynamic organization of cell shape, including cell motility. In migrating cell lamellipodia, nascent adhesions, consisting of clustered integrins and other cytoplasmic proteins such as focal adhesion kinase (FAK), ACTN, and vinculin (VCL) initially form. These are short-lived structures that either turnover rapidly in around 60 seconds, or mature to larger (approximately 1 μm in diameter) dot-like adhesions referred to as focal complexes that persist for several minutes. Focal complexes further grow centripetally into elongated focal adhesions and, concurrently, the associated actin stress fibers become thicker [26]. Polymerization of lamellipodial actin is catalyzed by actin nucleation promoting factors, WASP family verprolin-homologous protein (WAVE) family proteins and the actin-related protein 2/3 (Arp2/3) complex, which is also required for the assembly of nascent adhesions. Lamellipodial actin filaments, in concert with ACTNs, form precursors that serve as templates for the maturation of nascent adhesions within focal adhesions [27,28]. The maturation of nascent focal adhesion involves the participation of scaffolding proteins, namely, paxillin (PAX) and zyxin (ZYX), to form stable focal adhesions [7,26,29–31]. PAX seems to regulate the transition from nascent adhesions to focal complexes through multiple phosphorylated tyrosine residues of PAX. On the other hand, ZYX participation in focal adhesions is a relatively late event that occurs after focal complexes are formed. Thus, ZYX is thought to be involved in the formation and reorganization of fully mature, centripetal focal adhesions. In accordance, recent reports suggest the role of ZYX in stress fiber thickening in response to mechanical stresses [32]. ACTN1 is traditionally thought to be a linker between integrin complexes and stress fibers, since ACTN1 can directly bind to integrin β1, VCL, and ZYX [33,34]. However, the role of ACTN4, which exhibits a high amino acid similarity (86%) to ACTN1, has not been precisely tested, both in function and in protein-protein interactions in focal adhesion formation.

In this study, we demonstrate the opposing effects of non-muscle ACTNs in focal adhesion maturation in colorectal cancer cells. ACTN1 positively regulates focal adhesion formation, while ACTN4 induces destabilization and rapid turnover of focal adhesions in ACTN4-overexpressing cells. We also demonstrate, for the first time, a clear difference between the two ACTN isoforms in ZYX-binding. ACTN4 does not bind to ZYX, which likely underlies the observed high turnover rate of focal adhesions in ACTN4-overexpressing cells, while ACTN1 binds to ZYX as previously reported. ACTN4 failure to bind to ZYX closely associates with cancer pathogenesis, because compromised cell-substratum adhesion frequently leads to increased cancer cell motility and invasiveness.

## Materials and Methods

### Reagents and antibodies

The anti-ACTN1 antibody (H00000087) was purchased from Abnova (Taipei City, Taiwan). The anti-ACTN4 antibody (ALX-210-356) was purchased from Enzo Life Sciences (Plymouth Meeting, PA, USA). The anti-RhoA (sc-418) and anti-myosin regulatory light chain (MRLC) (sc-28329) antibodies were purchased from Santa Cruz Biotechnology Inc. (Santa Cruz, CA, USA). The anti-phospho-MRLC (T18/S19) antibody (#3674) and anti-His-tag antibody (#2365) were purchased from Cell Signaling Technology (Danvers, MA, USA). The anti-FLAG (F3165), anti-VCL (V9131), anti-ZYX (HPA004835), and pan-ACTN (A5044) antibodies were purchased from Sigma-Aldrich (St. Louis, MO, USA). The anti-PAX antibody (610051) was purchased from BD Biosciences (San Jose, CA, USA). The anti-phospho-SRC (Y418) antibody (44-660G) and anti-phospho-FAK (Y397) antibody (44-624G) were purchased from Life Technologies (*Carlsbad*, CA, USA). The anti-actin antibody (MAB1501) was purchased from EMD Millipore (Billerica, MA, USA). Rhodamine-labeled phalloidin was purchased from Life Technologies. Growth Factor Reduced Matrigel (#354230) was purchased from Corning (New York, NY, USA). Actin proteins purified from rabbit skeletal muscle (AKL99-A) were purchased from Cytoskeleton, Inc. (Denver, CO, USA).

### Cell culture

The human colorectal cancer cell lines DLD-1, HT-29, LoVo, NCI-H508, and Caco-2 were generous gifts from Dr. T. Azuma (Tokyo Dental College, Tokyo, Japan). The human colorectal cancer cell line SW480 was a generous gift from Dr. K. Nagano (The Institute of Medical Science, The University of Tokyo, Tokyo, Japan). DLD-1 and LoVo were originally purchased from JCRB Cell Bank (Osaka, Japan). HT-29, NCI-H508, Caco-2, and SW480 were from the American Type Culture Collection (ATCC, Manassas, VA, USA). All cell lines, except SW480, were cultured in Dulbecco's modified Eagle’s medium **(**DMEM; Wako, Osaka, Japan) supplemented with 10% fetal bovine serum (FBS), 4 mM L-glutamine, and penicillin/streptomycin. SW480 cells were cultured in Roswell Park Memorial Institute 1640 medium (RPMI 1640; Wako, Osaka, Japan) supplemented with 10% FBS. FreeStyle 293F cells were cultured in FreeStyle 293 Expression medium (Life Technologies).

### Plasmids and transfection

Full-length human ACTN1, ACTN4, and ZYX cDNAs were obtained by polymerase chain reaction (PCR) using DLD-1 cell cDNA as a template. The following deletion mutants of ZYX were amplified by PCR: ZYX-N (amino acids [aa] 1–51) and ZYX-C (aa 52–572). All cDNAs were sequenced and then subcloned into the pCMV2A, pCMV4A (Agilent Technologies, Santa Clara, CA, USA), pEGFP-N2, pEGFP-N3, pEGFP-C1, pmCherry-N1 (TAKARA BIO Inc., Otsu, Japan), pGEX-6P-1 (GE Healthcare, Piscataway, NJ, USA), and pFastBacHT A (Life Technologies). The GFP-vinculin (VCL) construct was previously described [35]. For ACTN stable expression in DLD-1 cells, cDNAs encoding GFP, ACTN1-GFP, and ACTN4-GFP were amplified by PCR and inserted into pIRESpuro3 vector (TAKARA BIO Inc.).

Plasmids were transfected in DLD-1 or SW480 cells using Lipofectamine 2000 reagent or in FreeStyle 293F cells with FreeStyle Max reagent according to the manufacturer’s instructions (Life Technologies).

### RNA interference (RNAi)

Small interfering RNAs (siRNAs) targeting human ACTN1 and ACTN4 (siGENOME SMARTpool) and siGENOME Non-Targeting Control siRNA Pool #2 were purchased from GE Dharmacon (Lafayette, CO, USA). siRNAs were transfected into DLD-1 cells with Lipofectamine RNAiMAX reagent (Life Technologies) at a final concentration of 10 nM. The transfected cells were cultured in a growth medium for 48–72 h before being subjected to western blotting or immunofluorescence staining.

### Generation of stable DLD-1-ACTNs-GFP cell lines

DLD-1 cells were transfected with pIRESpuro3-GFP,-ACTN1-GFP, or-ACTN4-GFP. Single colonies were isolated after 2 weeks of puromycin selection (1.5 μg/mL). Protein expression in cell lysates was assessed by immunoblotting with an anti-GFP antibody. The resultant cells were termed as DLD-1-GFP, DLD-1-ACTN1-GFP, or DLD-1-ACTN4-GFP.

### Purification of recombinant proteins

Human full-length ZYX and ZYX-C were cloned into the pCMV2B vector encoding an N-terminal FLAG tag and transfected into FreeStyle 293F cells. The cells were harvested by centrifugation 48 h after transfection, and the pellet was resuspended in immunoprecipitation (IP) buffer (20 mM Tris, pH 7.4, 100 mM NaCl, 0.5% NP-40, 1 mM ethylenediaminetetraacetic acid [EDTA], and 1 mM phenylmethylsulfonyl fluoride [PMSF]) supplemented with leupeptin and aprotinin. The cells were lysed by sonication and centrifuged at 100,000 ×*g* for 30 min. The supernatant was incubated with anti-FLAG M2 affinity gel (Sigma-Aldrich) for 2 h and then washed four times with IP buffer. The FLAG-ZYX-full and-ZYX-C bound to anti-FLAG M2 gel were immediately used for pull-down assay. Human ZYX-N was cloned into the pGEX-6P-1 vector. This construct was used to transform BL21 (DE3) pLysS *Escherichia coli* cells (Promega, Madison, WI, USA). The cells expressing the GST-fusion proteins were collected, resuspended in lysis buffer (40 mM Tris, pH 7.5, 150 mM NaCl, 0.5% Triton X-100, 5 mM EDTA, and 1 mM PMSF, and Complete Protease Inhibitor Cocktail Tablet [Roche, Basel, Switzerland]), lysed by sonication, and centrifuged at 12,000 ×*g* for 30 min. The supernatant was incubated with Glutathione Sepharose 4B (GE Healthcare) for 2 h and then washed with lysis buffer. Bound GST-ZYX-N was eluted with glutathione.

His-tagged human full length ACTN1 and ACTN4 were expressed in Sf9 cells by using the Bac-to-Bac Baculovirus Expression System (Life Technologies). Recombinant proteins were purified using Ni-NTA Agarose (QIAGEN, Venlo, Netherlands).

### Immunofluorescence microscopy

Cells were transfected with plasmids or siRNAs and re-plated on Matrigel-coated coverslips after 24–48 h of transfection. The cells were allowed to adhere and spread for 24 h, and then fixed with 4% paraformaldehyde for 10 min at room temperature, followed by permeabilization using 0.2% Triton X-100 in phosphate-buffered saline (PBS) for 5 min. The coverslips were washed twice with PBS and incubated with the primary antibodies for 2 h at room temperature, and then treated with secondary antibodies for 1 h at room temperature. Alexa Fluor 568 Goat Anti-Rabbit IgG (H+L) Antibody and Alexa Fluor 647 Goat Anti-Mouse IgG (H+L) Antibody (both from Life Technologies) were used as secondary antibodies. The coverslips were mounted with PermaFluor Aqueous Mounting Medium (Thermo Scientific, Waltham, MA, USA) and observed using a FluoView 1000-D confocal microscope (Olympus, Tokyo, Japan).

### Western blotting

All cells used for western blotting were seeded in plastic dishes precoated with Matrigel. The cells were lysed with Laemmli sample buffer, sonicated briefly to sever DNA, and boiled at 95°C for 5 min. Western blotting was done by standard procedures with detection by alkaline phosphatase-conjugated goat anti-mouse or-rabbit IgG secondary antibodies (Promega). ImageJ software (National Institutes of Health, Bethesda, MD, USA) was used to quantify band intensities.

### Quantification of focal adhesions and stress fibers

Focal adhesions were quantified as follows. Cells were fixed and stained with anti-VCL antibody and rhodamine-phalloidin, or with anti-PAX and anti-ZYX antibodies. Images were obtained by confocal microscopy and thresholded for three times the background. Focal adhesion numbers were quantified by counting VCL or PAX plaques. To quantify focal adhesion maturity, PAX- and ZYX-positive plaques per unit area (DLD-1 cells) or per cell (SW480 cells) were counted. Eight to eighty cells obtained from three independent experiments were analyzed. The ratio of ZYX-positive to PAX-positive (total) plaques was calculated for each cell and then averaged between cells. To quantify focal adhesion size, the mean area of PAX-positive plaques was calculated for each cell and then averaged between cells. All the image processing was done using ImageJ software.

Stress fiber formation was quantified as follows. Cells transfected with control, ACTN1, and ACTN4 siRNAs, or cells expressing GFP, ACTN1-GFP, or ACTN4-GFP were stained with anti-VCL antibody and rhodamine-phalloidin and imaged by confocal microscopy under the same acquisition settings. Phalloidin signals were thresholded at the same level and the percentage of cells forming discernable stress fibers connected to VCL plaques in at least one end was quantified. The experiments were repeated three times. At least 23 cells were scored for each experiment.

### Live-cell imaging of DLD-1 cells and turnover analysis

DLD-1 cells transfected with GFP-VCL and mCherry, ACTN1-mCherry, or ACTN4-mCherry were re-plated on Matrigel-coated, 35-mm glass-bottom dishes (IWAKI, Tokyo, Japan) 24 h after transfection. About 16 h after re-plating, the cells were observed for more than 2 h by total internal reflection fluorescence (TIRF) microscopy (Olympus). Before collection of the images, the medium was replaced with a CO_2_-independent medium (Life Technologies) supplemented with 10% FBS and 4 mM l-glutamine. The images were taken as one frame every minute for 120 min. The imaged adhesions fit the criteria in that they were localized to a protruding lamellipodia. A sequence of kymographs was obtained using ImageJ software. The time-lapse change in the fluorescence intensity of GFP-VCL was plotted using Microsoft Excel (Microsoft Corporation, Redmond, WA, USA), and the rates of focal adhesion assembly, stability, and disassembly were determined as previously described [28]. These measurements were carried out for 2–10 adhesions per cell in 8–10 cells for each condition.

### Matrigel invasion assay

The invasion assay was performed as previously described [36], with some modifications. DLD-1-GFP, DLD-1-ACTN1-GFP, and DLD-1-ACTN4-GFP cells (2.0 × 10^5^ cells in serum-free DMEM) were seeded in the upper compartment of BD BioCoat Matrigel Invasion Chamber (BD Biosciences) and incubated with 10% FBS DMEM in the bottom well and serum-free medium in the top chamber. After 26 h, the cells were fixed with 3.7% formaldehyde in PBS for over 30 min. Cells were washed with PBS and the invaded GFP-positive cells on the undersurface of the filter membrane were observed by confocal microscopy. The cells in six randomly selected microscopic fields from duplicate chambers were counted in three independent experiments. SW480 cells were transfected with GFP or ACTN1-GFP plasmids. After 24 h, cells (5.0 × 10^5^ cells) were seeded in the top chamber and incubated with 10% FBS RPMI 1640 in the bottom well and serum-free medium in the top chamber.

### Pull-down assay

GST-ZYX-N proteins bound to Glutathione Sepharose 4B were incubated with purified His-ACTN1 or His-ACTN4 proteins in binding buffer (lysis buffer plus 1.0% bovine serum albumin [BSA]) for 2 h, washed thrice with binding buffer, and bound ACTNs were analyzed by SDS-PAGE. FLAG-ZYX-full and FLAG-ZYX-C proteins bound to anti-FLAG M2 affinity gel were mixed with purified His-ACTN1 or His-ACTN4, incubated for 2 h in IP buffer, and bound ACTNs were analyzed. The amounts of active RhoA were quantified in pull-down assays with GST-Rhotekin-RBD, as previously described [36]. The signal intensities were measured using ImageJ software.

### Statistical analysis

For multiple comparisons, *P* values were generated by one-way analysis of variance (one-way ANOVA) using the Dunnett’s test for multiple comparisons. For two group analyses, Student's *t*-test was used. Analyses were performed using GraphPad Prism 6 software (GraphPad Software, Inc., San Diego, CA, USA).

## Results

### ACTN1 and ACTN4 expression in colon cancer cells

**Fig 1:**
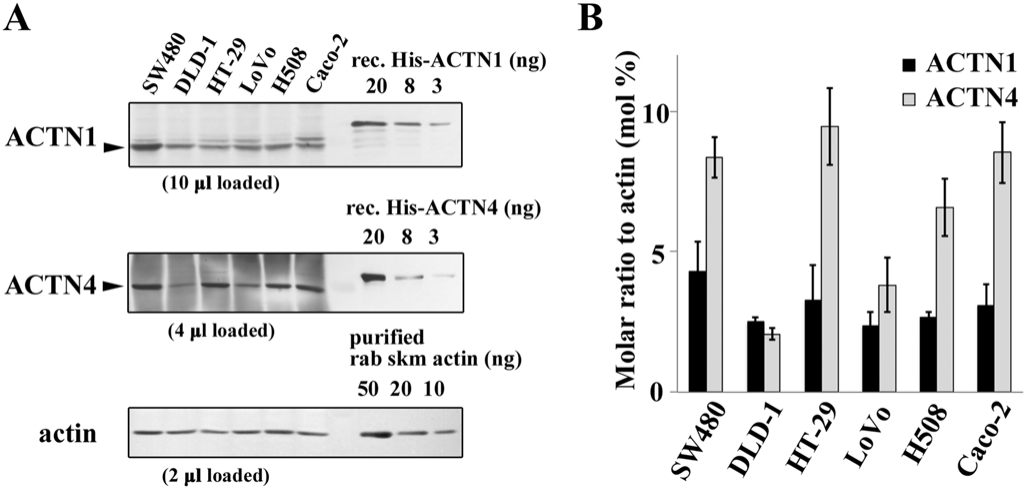
Expression of α-actinin-1 (ACTN1) and ACTN4 in human colon cancer cell lines. **(A)** Western blot analysis of ACTN1, ACTN4, and actin in human colon cancer cell lines. ACTN1 and ACTN4 protein levels were measured in comparison with recombinant His-tag ACTN1 and ACTN4 proteins used as external standards. Actin was used as a loading control and purified rabbit skeletal muscle actin was used as an external standard. **(B)** Band intensities were quantified, and the molar ratios of ACTNs were calculated and represented as mol% against actin. The data represent the mean ± SD of three independent experiments.

ACTN4 was reported to enhance cell motility and promote lymph node metastasis of colorectal cancer [13]. However, it is not clear whether these effects are attributed to ACTN4 isoform-specific functions or whether ACTN1 classical isoform is capable of inducing cancerous phenotypes as ACTN4 does. We first quantified the relative expression levels of endogenous ACTN1 and ACTN4 proteins in several colon cancer cell lines using purified recombinant proteins as external controls (Fig 1A). ACTN4 expression was higher than that of ACTN1 in all cells, except DLD-1 cells. DLD-1 cells expressed ACTN4 at a low, but similar, level than that of ACTN1 (Fig 1B). Thus, in order to clarify the differential function of ACTN1 and ACTN4, we used DLD-1 cells because the effects of ectopic expression or silencing of ACTNs were detectable.

### Differential roles of ACTN1 and ACTN4 in the formation of focal adhesions

**Fig 2:**
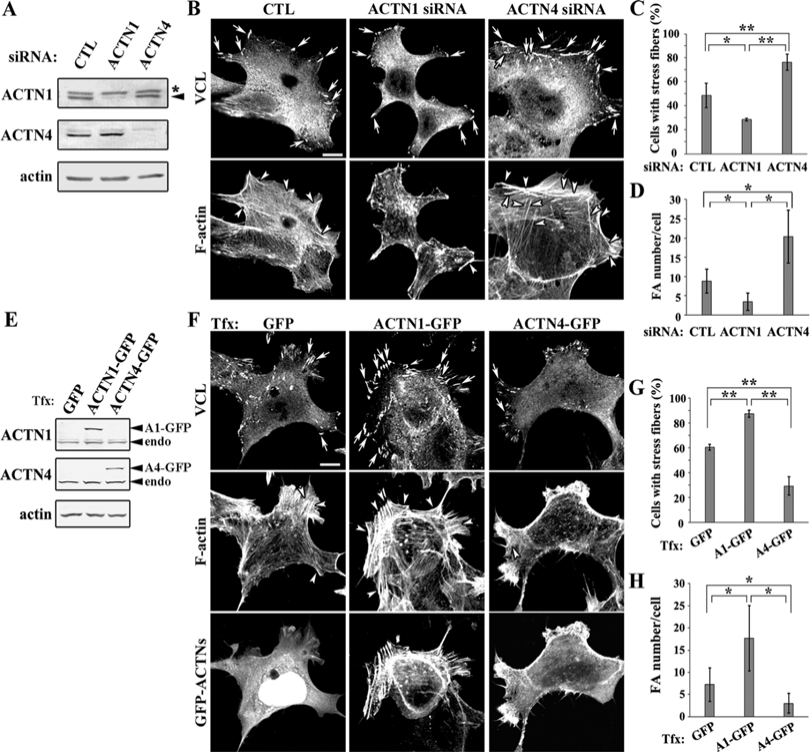
Effect of ACTN1 and ACTN4 on DLD-1 cell morphology. **(A)** ACTN1 and ACTN4 expression was silenced by siGENOME SMARTpool siRNA in DLD-1 cells. Efficient knockdown was confirmed by western blotting using ACTN isoform-specific antibodies. The arrowhead indicates endogenous ACTN1. The asterisk indicates a non-specific crossreacting band. **(B)** Cells transfected with the indicated siRNAs were stained for vinculin (VCL) and F-actin and observed by confocal microscopy. The arrows and arrowheads indicate focal adhesions and stress fibers, respectively. Scale bar = 10 μm. **(C)** The percentages of siRNA-treated cells forming stress fibers are graphed. Data represent the mean ± SD of three independent experiments. *P < 0.05, **P < 0.01. **(D)** The focal adhesion numbers per cell of siRNA-treated cells were graphed. Data represent the mean ± SD of 71 cells for control, 73 cells for si-ACTN1 cells, and 55 cells for si-ACTN4 cells. *P < 0.05. **(E)** Transient expression of ACTN1-GFP and ACTN4-GFP in DLD-1 cells was confirmed by western blotting using ACTN isoform-specific antibodies. **(F)** GFP, ACTN1-GFP, and ACTN4-GFP were expressed in DLD-1 cells, which were then stained for VCL and F-actin and observed by confocal microscopy. The arrows and arrowheads indicate focal adhesions and stress fibers, respectively. Scale bar = 10 μm. **(G)** The percentages of transfected cells forming stress fibers are graphed. Data represent the mean ± SD of three independent experiments. Tfx indicates transfection. **P < 0.01. **(H)** The focal adhesion numbers per cell of cells expressing GFP, ACTN1-GFP, and ACTN4-GFP were graphed. Data represent the mean ± SD of 130 cells for GFP, 52 cells for ACTN1-GFP, and 73 cells for ACTN4-GFP. *P < 0.05.

ACTN1 and ACTN4 expression in DLD-1 cells was silenced by siRNA (Fig 2A). ACTN1 knockdown decreased the number of stress fibers and focal adhesions. However, ACTN4 knockdown unexpectedly increased stress fibers and focal adhesions (Fig 2B–2D). These results show that both ACTN1 and ACTN4 are involved in the formation of stress fibers and focal adhesions in DLD-1 cells, but clearly in opposite ways: ACTN1 enhances it, while ACTN4 suppresses it. The contrasting roles of ACTNs were also observed by ectopic expression of ACTN1-GFP and ACTN4-GFP in DLD-1 cells (Fig 2E). ACTN4 overexpression suppressed the formation of stress fibers and focal adhesions (Fig 2F–2H). On the other hand, ACTN1 overexpression induced the development of stress fibers and focal adhesions.

Major signaling molecules that transmit information from adhesion plaques to actin stress fibers include myosin II, FAK, SRC family kinases, and Rho family GTPases. Thus, we examined the proteins that are involved in the formation of focal adhesions by western blotting in cells in which ACTN expression was silenced by siRNA (S1 Fig). Phosphorylation of myosin regulatory light chains (MRLC) on threonine 18 and serine 19, and tyrosine phosphorylation of FAK and SRC were not altered. RhoA activity was decreased specifically in ACTN1 siRNA-treated cells. Although decrease in myosin II activity in ACTN1 or ACTN4 knockdown cells was previously reported [14,17,37], we could not detect significant changes in MRLC phosphorylation. Additionally, the decrease observed in RhoA activity was modest (less than 40% decrease for ACTN1 siRNA). These changes did not correlate with phenotypic alterations in the formation of stress fibers and focal adhesions observed in ACTN knockdown cells in Fig 2B–2D and 2F–2H. In addition, the activity of regulators of nascent adhesion formation, i.e. FAK and SRC, was similar in control and ACTN1/4 siRNA-treated cells, suggesting that the alterations of stress fiber formation and focal adhesion numbers in response to manipulation of ACTN expression could be attributed to the differential activities of late-acting molecules in the stepwise maturation of focal adhesions such as regulators of focal complex stability or regulators of centripetal focal adhesion assembly.

### ACTN4 enhances focal adhesion turnover rate

**Fig 3:**
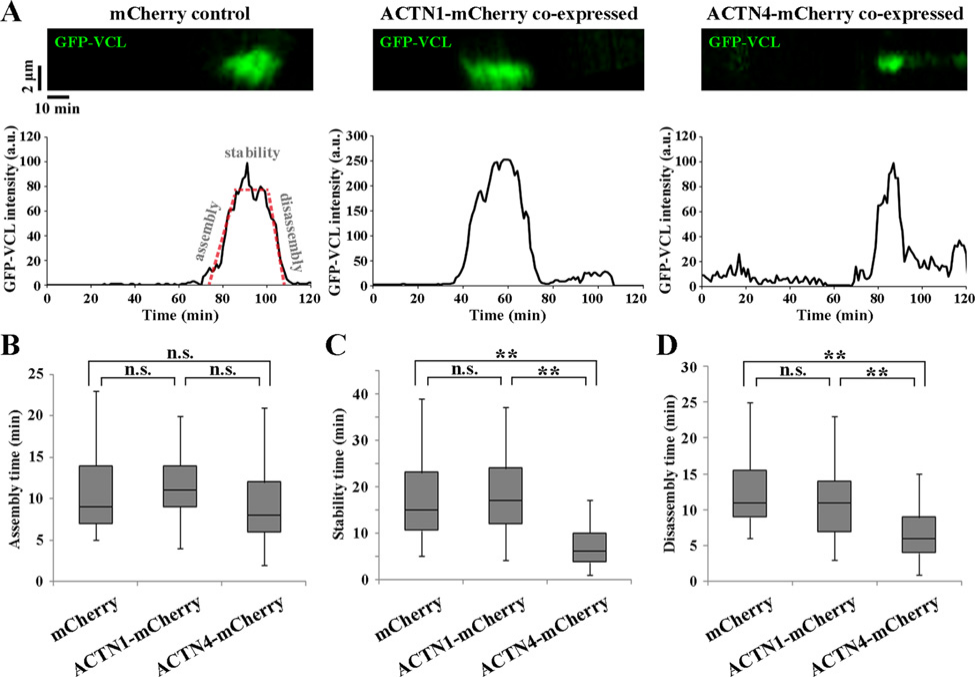
Turnover rate of focal adhesions in mCherry-, ACTN1-mCherry-, or ACTN4-mCherry-overexpressing DLD-1 cells. **(A)** mCherry, ACTN1-mCherry, or ACTN4-mCherry were co-expressed with GFP-VCL and VCL time lapse image was observed by total internal reflection fluorescence (TIRF) microscopy to trace the assembly and disassembly of focal adhesions. Upper panels show representative kymographs of GFP-VCL fluorescence along 5-μm long lines drawn in regions of extending lamellipodia. Fluorescence intensity in the upper panel was plotted against time and shown as a graph to the bottom of respective kymographs. The turnover phases of focal adhesion assembly, stability, and disassembly are presented as red dashed lines in the mCherry vector graph. **(B–D)** Duration of each turnover phase exemplified in the left graph in A was analyzed and quantified from 51 adhesions for mCherry-expressing cells, 57 adhesions for ACTN1-mCherry-expressing cells, and 64 adhesions for ACTN4-mCherry-expressing cells. Data are presented as box and whisker plots with boxes representing 25th–75th percentile range and whiskers representing 10th–90th percentile range. n.s., not significant, **P < 0.01.

Next, we co-expressed ACTN1-mCherry or ACTN4-mCherry with GFP-VCL and observed VCL time-lapse images by TIRF microscopy to trace the assembly and disassembly of focal adhesions. As shown in Fig 3A, S1 Movie, S2 Movie, and S3 Movie, the turnover rate of focal adhesions in ACTN4-expressing cells appeared faster than that of control or ACTN1-expressing cells. The time required for a single adhesion to be disassembled was 34 min on average in control mCherry-expressing cells, indicating that the adhesion demarcated by GFP-VCL signal is presumed to reach a mature focal adhesion. The lifetime of such focal adhesions could be divided into three steps; assembly, stability, and disassembly (Fig 3A). We measured the time required for each step (Fig 3B–3D). In ACTN4-expressing cells, the time required for stabilization and disassembly was shorter than in control or ACTN1-expressing cells. These results demonstrate that in ACTN4-expressing cells, the lifetime of focal adhesions is shortened due to an accelerated disassembly rate, suggesting that focal adhesions are destabilized in an isoform-specific manner by ACTN4 overexpression.

### ZYX recruitment to focal complexes is impaired in ACTN4-expressing cells

**Fig 4:**
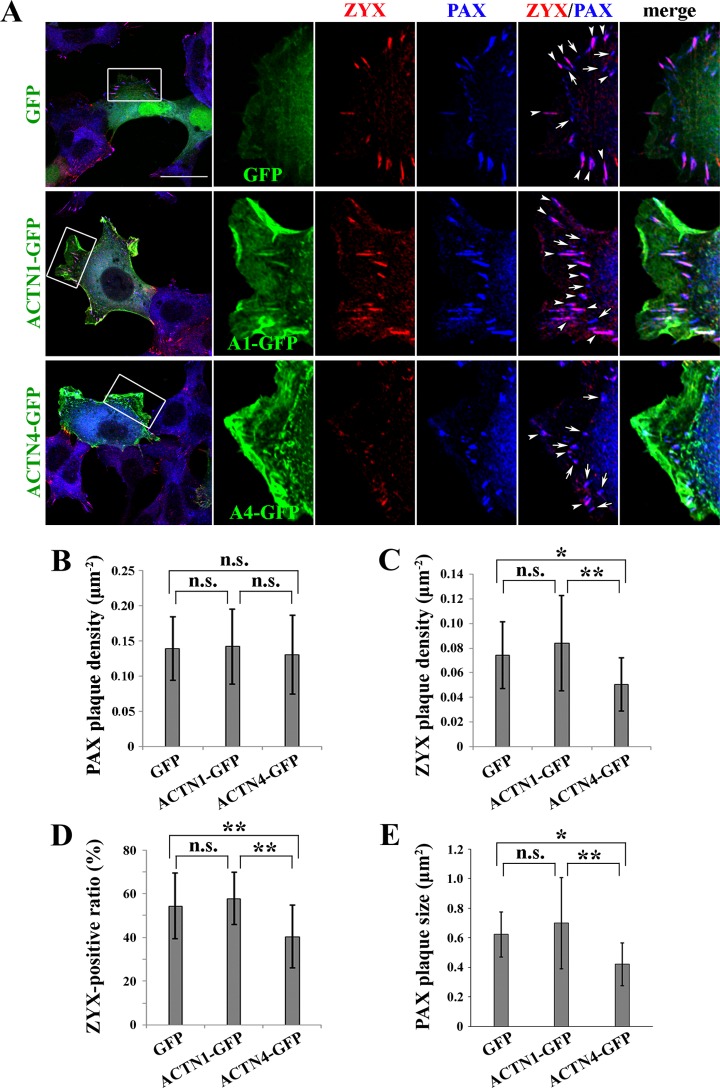
ZYX recruitment to focal complexes in ACTN1- or ACTN4-expressing DLD-1 cells. **(A)** GFP, ACTN1-GFP, and ACTN4-GFP (shown in green) were expressed in DLD-1 cells, which were stained for PAX (blue) and ZYX (red) proteins and imaged under a confocal fluorescence microscope. Boxed regions in the leftmost column indicate the peripheral lamellae used for PAX and ZYX plaque quantification. A series of enlarged images of the boxed regions are shown to the right. The arrows and arrowheads indicate PAX-positive and PAX/ZYX-double-positive plaques, respectively. Scale bar = 30 μm. **(B–C)** Numbers of PAX and ZYX plaques per unit area of peripheral lamellae were counted in GFP-, ACTN1-, or ACTN4-expressing cells. Eight to sixteen cells were analyzed per group. Error bars refer to standard deviations between cells. n.s., not significant, *P < 0.05, **P < 0.01. **(D)** Ratio of ZYX-positive to PAX-positive plaques, i.e., total plaque adhesions. Error bars refer to standard deviations between cells. n.s., not significant, **P < 0.01. **(E)** PAX plaque size in GFP-, ACTN1- or ACTN4-expressing cells was measured in at least 171 adhesions from 8–11 cells per group. Error bars refer to standard deviations between cells. n.s., not significant, *P < 0.05, **P < 0.01.

Given that focal adhesions are destabilized by ACTN4 expression in DLD-1 cells, we hypothesized that ACTN4 inhibits the function of proteins involved in the transformation of focal complexes to centripetal focal adhesions. In particular, we examined the localization of ZYX, which is reported to specifically localize in mature centripetal focal adhesions [30]. We used PAX as a general marker of focal adhesions throughout maturation. As shown in the upper row of Fig 4A, GFP-expressing control DLD-1 cells formed PAX-positive plaques in the lamellar cell periphery. These plaques were assumed to correspond to both focal complexes and centripetal focal adhesions. Among these plaques, mature centripetal focal adhesions could be distinguished based on ZYX colocalization. The average numbers of PAX- and ZYX-positive plaques in control cells were 0.14 and 0.073 per square micrometer, respectively (Fig 4B and 4C). Hence, approximately half of the focal adhesions were ZYX-positive and were thus considered to be mature in control cells. ACTN1-GFP- and ACTN4-GFP-expressing cells formed similar numbers of PAX plaques as observed in control cells, suggesting that ACTNs have little influence on the formation of focal complexes (Fig 4B). In contrast, both the number and ratio of ZYX-positive plaques in ACTN4-expressing cells were significantly reduced to 0.049 plaques/μm^2^ and 40%, respectively, compared to those of control and ACTN1-expressing cells (Fig 4C and 4D). In addition, the predominant shape of focal adhesions in ACTN4-expressing cells was changed to a round, dot-like structure in contrast to the elongated centripetal adhesions that were frequently observed in control and ACTN1-expressing cells (see arrows in Fig 4A, bottom row). The change in shape was reflected in the size of PAX-positive plaques that became smaller in ACTN4-expressing cells (Fig 4E). These results show that focal complexes cannot reach the matured centripetal focal adhesions in ACTN4-expressing cells. In addition, ACTN4 specifically inhibits the proper recruitment of ZYX to focal complexes, which may explain the phenotypic differences between ACTN1- and ACTN4-expressing cells.

### ACTN4 does not bind to ZYX

**Fig 5:**
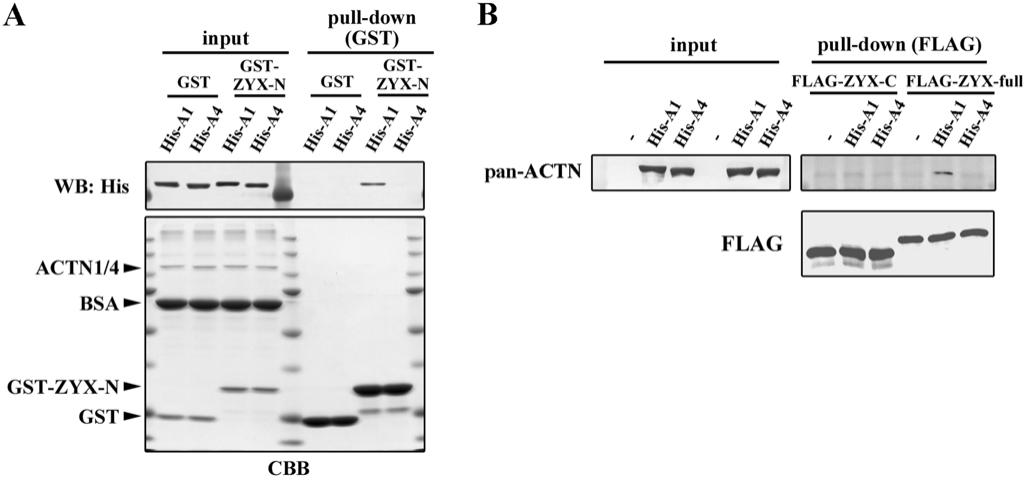
ZYX binding to ACTN1 and ACTN4. **(A)** Pull-down assay showing that a putative ACTN-binding site, GST-ZYX-N (aa 1–51), binds specifically to His-ACTN1, but not to His-ACTN4. Bound His-ACTN1 or His-ACTN4 was analyzed by western blotting using anti-His-tag antibody. Equal amounts of input proteins were confirmed by SDS-PAGE followed by Coomassie blue staining (CBB). **(B)** FLAG-ZYX-full (aa 1–572) or FLAG-ZYX-C (aa 52–572) bound to anti-FLAG M2 beads were mixed either with His-ACTN1 or His-ACTN4 proteins, or without ACTN proteins (denoted by “-”). Bound His-ACTN1 or His-ACTN4 was analyzed by western blotting using anti-pan-actinin antibody. Note that the faint bands detected in lanes 1 and 4 of the pull-down blot represent non-specific background signals of pan-actinin antibody. Equal amounts of ZYX proteins were confirmed by western blotting using anti-FLAG antibody.

Next, we investigated whether the impaired recruitment of ZYX to focal adhesions in ACTN4-expressing cells was due to a defect in ZYX binding to ACTN4. The binding site of ZYX to ACTN1 was previously determined [34]. According to this report, the N-terminal region (aa 1–51) of ZYX (ZYX-N) is a binding site for ACTN1. We carried out a pull-down assay by mixing GST-ZYX-N protein and His-ACTN1 or His-ACTN4 protein. As shown in Fig 5A, GST-ZYX-N bound to His-ACTN1, but not to His-ACTN4. Furthermore, we tested whether the full-length ZYX protein exhibits selective binding to ACTN1. We carried out a pull-down assay using purified FLAG-tagged full-length ZYX protein (ZYX-full) or an N-terminal deletion mutant of ZYX (ZYX-C; aa 52–527) that lacks the ACTN1-binding sequence (Fig 5B). Binding of the full-length ZYX to ACTN4 was not observed, while its binding to ACTN1 was clearly detected. In contrast, ZYX-C did not bind to either ACTN1 or ACTN4, demonstrating that the ZYX N-terminal sequence in the full-length protein is responsible for differential binding to ACTNs. Altogether, these data suggest that ACTN4 does not bind as effectively to ZYX as does ACTN1, which presumably underlies the impaired recruitment of ZYX during focal adhesion maturation of ACTN4-expressing cells.

### ACTN4 enhances DLD-1 cell invasion

**Fig 6:**
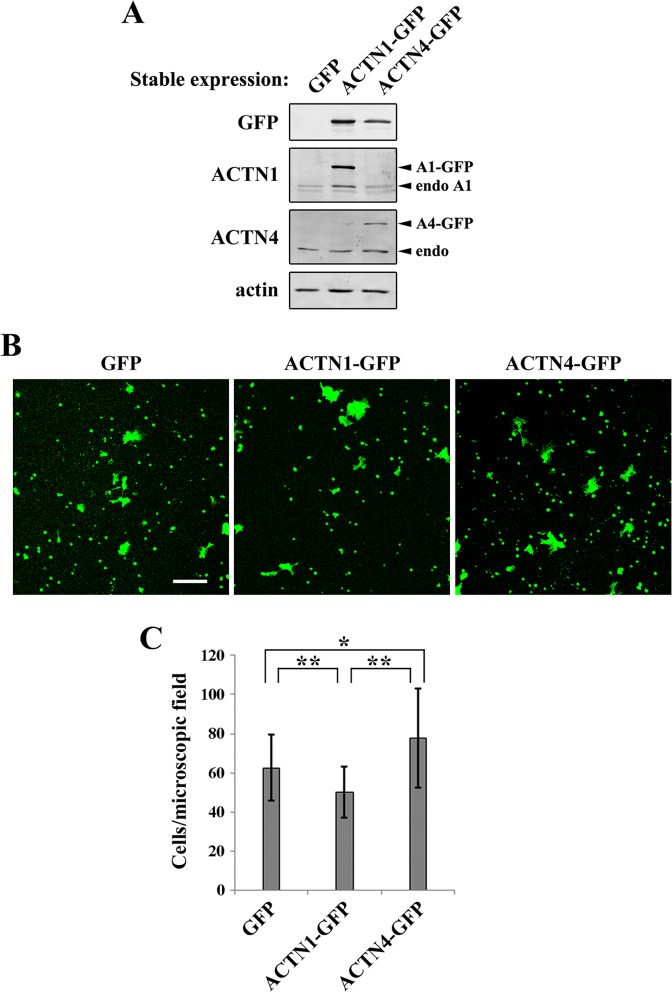
Invasion of DLD-1 cells expressing GFP, ACTN1-GFP, and ACTN4-GFP. **(A)** Stable expression of GFP, ACTN1-GFP, and ACTN4-GFP in DLD-1 cells was examined by western blotting using the antibodies indicated to the left of each blot. **(B)** The invasion assay was performed using GFP-, ACTN1-GFP-, or ACTN4-GFP-expressing stable cell lines. Cells were seeded into Matrigel invasion chambers and allowed to invade into the Matrigel for 26 h. Then, the invaded cells were fixed and imaged by examining the GFP signals on the undersurface of the chamber using fluorescence confocal microscopy. Scale bar = 100 μm. **(C)** The GFP-positive cells shown in (B) were quantified. At least six microscopic fields obtained from duplicated chambers were used for quantification in each experiment. The results represent the mean ± SD of three independent experiments. *P < 0.05, **P < 0.01.

To investigate whether the above-described functional differences of ACTN1 and ACTN4 contribute to cancer-associated phenotypes, the effect of ACTN1 and ACTN4 on DLD-1 cell invasion was examined by Matrigel invasion assay. Since the transient transfection efficiency of DLD-1 was low, we established stable cell lines that ectopically expressed ACTN1-GFP or ACTN4-GFP (Fig 6A). Compared to the control GFP-expressing line, ACTN1-GFP-expressing cells were less invasive, as measured by the number of GFP-positive cells that had moved through the Matrigel and adhered onto the bottom surface of the chamber (Fig 6B and 6C). By contrast, ACTN4-GFP-expressing cells were more invasive than control and ACTN1-GFP-expressing cells (Fig 6C). These results show that increased expression of ACTN4 enhances DLD-1 cell invasion in an isoform-specific manner, which is consistent with the previous observations that ACTN4 expression correlates with malignant phenotypes of colon cancer cells.

### ACTN1 suppresses the invasion of ACTN4-overexpressing SW480 cells

In DLD-1 cells, we demonstrated the opposing roles of ACTN1 and ACTN4 in stress fiber formation, focal adhesion formation, and Matrigel invasion. These differences between isoforms likely arise from the lack of ACTN4 binding to ZYX. It is possible that ACTN1 and ACTN4 could compete with each other to occupy the focal complexes, and that ACTN4 overexpression could displace ACTN1 from focal complexes, rendering them incompetent to recruit ZYX for focal adhesion maturation. To corroborate this possibility, we used SW480 cells that expressed higher endogenous levels of ACTN4 protein than ACTN1 (Fig 1). ACTN1-GFP was ectopically overexpressed in SW480 cells to reduce the relative ACTN4 amount, and changes in focal adhesion components and invasion ability were assessed.

**Fig 7:**
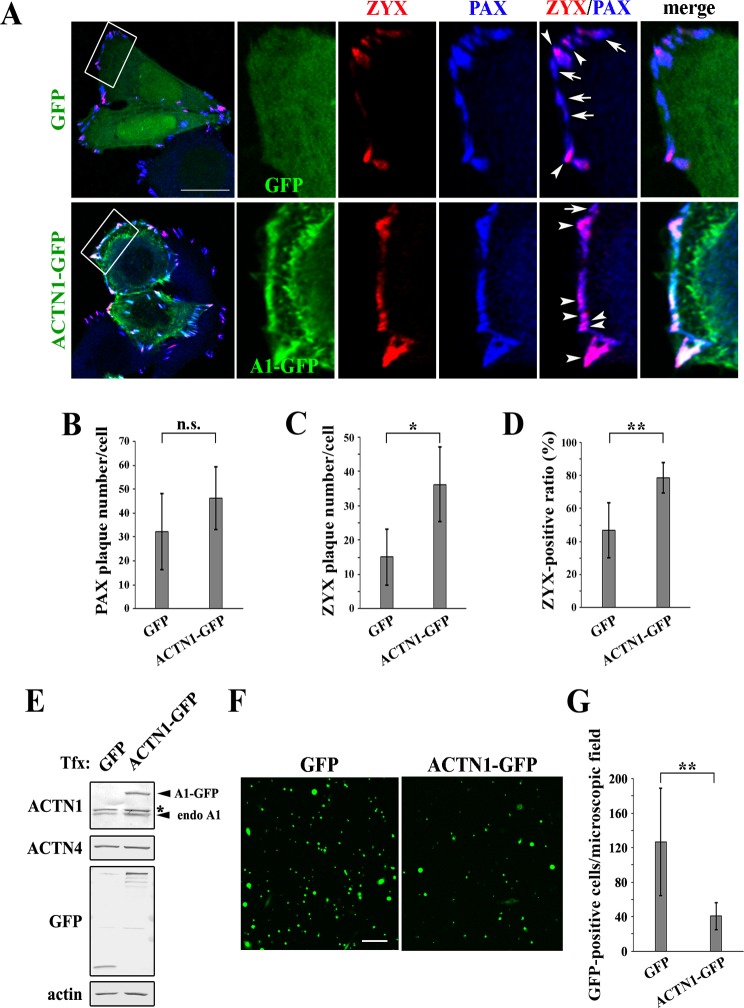
ACTN1 expression suppresses SW480 cell invasion. **(A)** SW480 cells expressing GFP or ACTN1-GFP (shown in green) were stained for PAX (blue) and ZYX (red) proteins and imaged as described for Fig 4A. Boxed regions in the leftmost column are enlarged and shown to the right. The arrows and arrowheads indicate PAX-positive and PAX/ZYX-double-positive plaques, respectively. Scale bar = 20 μm. **(B–C)** Numbers of PAX and ZYX plaques per cell were counted in GFP- or ACTN1-GFP-expressing cells. At least 66 cells were analyzed per group. Error bars refer to standard deviations between cells. n.s., not significant, *P < 0.05. **(D)** Ratio of ZYX-positive to PAX-positive plaques. Error bars refer to standard deviations between cells. **P < 0.01. **(E)** SW480 cells were transiently transfected with GFP or ACTN1-GFP, and protein expression was analyzed by western blotting using the antibodies indicated to the left of each blot. Tfx indicates transfection. The asterisk indicates a non-specific crossreacting band. **(F)** The invasion assay was performed using GFP- or ACTN1-GFP-expressing SW480 cells as described in the legend to Fig 6B. Invaded cells were fixed and imaged by GFP fluorescence. Scale bar = 100 μm. **(G)** The GFP-positive cells shown in (F) were quantified as described in the legend to Fig 6C. **P < 0.01.

GFP-expressing control SW480 cells formed PAX-positive focal adhesions at about 32 plaques per cell on average, 47% of which were ZYX-positive and thus thought to be mature centripetal focal adhesions (Fig 7A–7D). ACTN1-expressing cells formed a comparable but slightly increased number of PAX-positive plaques compared to control cells (Fig 7B). However, the number of ZYX-positive plaques was significantly increased (Fig 7A arrowheads, and Fig 7C), and the frequency of ZYX-positive plaques was increased to 79% (Fig 7D). These results suggest that ACTN1 expression can restore the recruitment of ZYX to focal complexes in ACTN4-overexpressing SW480 cells.

Although the invasion of DLD-1 cells was suppressed by ectopic ACTN1 expression, the suppressive effect was minimal, which was likely due to the low endogenous level of ACTN4 expression in these cells (Fig 6). Thus, it was presumed that Matrigel invasion should be more potently suppressed by ectopic ACTN1 expression for ACTN4-overexpressing SW480 cells than for DLD-1 cells. We first confirmed that the transfection efficiency of SW480 cells was similar between GFP and ACTN1-GFP (S2 Fig), and that the ACTN1-GFP expression levels were higher than those of endogenous ACTN1 (Fig 7E). As the efficiency of transient transfection was as high as 40%, we counted only GFP-positive cells for quantification of invaded cells to precisely reflect the effect of transgene expression. As shown in Fig 7F and 7G, SW480 cell invasion was strongly suppressed by ACTN1-GFP expression, with a greater than 60% reduction of invaded cells compared to control cells. The results from both DLD-1 cells and SW480 cells indicate that the biased expression of ACTN isoforms dictates the invasive property of colon cancer cells; higher relative expression of ACTN4 over ACTN1 tends to favor invasive phenotypes. Our finding that ACTN1 and ACTN4 show differential affinities for ZYX may provide the molecular mechanism underlying the phenotypic outcome of ACTN overexpression in colon cancer cells.

## Discussion

Elevated levels of ACTN4 protein expression are found in a number of human cancers and associated with cancer malignancies and poor patient outcome. However, it is not yet clear whether these characteristics of ACTN4 are derived from its isoform-specific functions, which is mainly due to a lack of knowledge about the molecular differences between the two non-muscle isoforms ACTN1 and ACTN4. Thus, we set out to investigate the molecular functions of these two isoforms. Prior to conducting this study, we examined the biochemical properties of ACTN1 and ACTN4 using purified His-tagged proteins. However, we could not detect clear differences in well-known ACTN functions, including actin-binding, actin-bundling, and phosphatidylinositol (4,5)-bisphosphate (PI(4,5)P_2_)-binding. In this study, we found that ZYX specifically binds to ACTN1, but not to ACTN4, demonstrating for the first time that putative ACTN-binding partners show isoform selectivity (Fig 5). We also showed functional differences between ACTN1 and ACTN4 during focal adhesion maturation processes, which could be accounted for by the lack of binding of ACTN4 to ZYX. During normal focal adhesion maturation, focal complexes contain sufficient numbers of ACTN1 molecules to recruit ZYX, which is required for proceeding through subsequent maturation steps. If ACTN4 protein levels are elevated, for example, due to copy number variations in cancer cells [19,20,24], the focal complexes consequently become predominantly occupied by ACTN4. These ACTN4-enriched focal complexes are incompetent to recruit ZYX, and thus become unstable and prone to high turnover, which leads to increased cell motility. In accordance with this notion, we observed that ectopic expression of ACTN1 in ACTN4-overexpressing SW480 cells restored the recruitment of ZYX to the focal adhesions and suppressed SW480 Matrigel invasion (Fig 7).

It should be noted, however, that the effect of ACTN4 overexpression is likely not as simplistic as described above. For example, we observed a modest reduction in RhoA activity specifically in ACTN1 siRNA-treated DLD-1 cells (S1 Fig). Given the established role of RhoA in focal adhesion and stress fiber formation, such ACTN1-mediated RhoA activation at focal complexes may contribute to focal adhesion maturation in parallel to ZYX recruitment. Another complicating factor that should be taken into account is that ACTN1 and ACTN4 can form a heterodimer [25]. In this study, we examined *in vitro* interactions between ZYX and ACTNs using purified proteins, in which ACTN heterodimerization does not occur. However, in living cells, ACTN1/ACTN4 heterodimers form a substantial proportion of the total ACTN dimers. Therefore, further detailed analysis of the interaction between ACTN and ZYX is necessary to determine whether the heterodimer is able to bind to ZYX. This will reveal the precise contribution of each isoform in focal adhesion formation and the relevance of elevated levels of ACTN4 in cancer cells. Lastly, differential tyrosine phosphorylation between ACTN1 and ACTN4 has been reported. ACTN1 is phosphorylated by FAK at Y12 and is dephosphorylated by protein-tyrosine phosphatase 1B (PTP1B) [38,39], whereas ACTN4 is phosphorylated at Y4, Y31, and Y265 by as-yet-unknown upstream kinases [40]. Importantly, phosphorylation of Y12 decreases the ability of ACTN1 to bind to actin [38]. Y4 and Y31 phosphorylation also decreases ACTN4 binding to actin, whereas Y265 phosphorylation increases its affinity for actin [40]. Thus, the ability of ACTN1 and ACTN4 to bind to actin is differentially regulated by tyrosine phosphorylation and by regulatory kinases and phosphatases that localize at focal adhesions, which would undoubtedly affect focal adhesion formation. In addition to ZYX binding, all of the above-described differences between ACTN1 and ACTN4 could contribute to generate the contrasting functions of these isoforms in regulating cell behaviors.

Development of metastatic cancer is associated with increased local cancer cell invasion that is mediated in part by alterations in cell-substrate adhesion. In particular, two non-receptor tyrosine kinases, FAK and SRC, which play central roles in regulating focal adhesion formation and turnover, are frequently dysregulated in cancer progression [41,42]. FAK is recruited at nascent focal adhesions and is activated by autophosphorylation at tyrosine 397. Activated FAK binds to SRC and the Rho guanine nucleotide exchange factor (GEF) ARHGEF28 (also known as p190RhoGEF and RGNEF) to facilitate focal adhesion maturation [43,44]. SRC activity is correlated with colon cancer disease stage [45], and cell lines with elevated SRC activity are more motile and invasive *in vitro* [46]. Moreover, ARHGEF28 promotes the local invasion of orthotopic colon carcinomas in mice [47]. These observations indicate a close relationship between cell-substratum alterations and the malignant progression of colon cancer. Our results support this link, in demonstrating an ACTN4-mediated increase in focal adhesion turnover rate and Matrigel invasion. However, the levels of tyrosine phosphorylation of FAK Y397 and SRC Y418 were not changed by ACTN4 knockdown (S1 Fig). This suggests that the rapid turnover observed in ACTN4-expressing cells does not involve alterations in the FAK-SRC signaling axis. Thus, elevation of ACTN4 protein expression and ancillary ZYX inhibition in colon cancer cells provides another mechanistic basis for cancer progression, while underscoring the importance of focal adhesion regulation in colon cancer. Therefore, further in-depth studies on the roles of focal adhesion in colon cancer progression would be valuable to better understand the disease and to develop novel and effective strategies for treatment.

## Supporting Information

S1 Fig Effect of ACTN1 and ACTN4 expression on signaling proteins involved in focal adhesion formation. **(A)** Western blotting of myosin regulatory light chains (MRLC), phosphorylated MRLC (pMRLC), phosphorylated focal adhesion kinase (pFAK), phosphorylated SRC (pSRC), VCL, ACTN1, ACTN4 and actin in control, ACTN1, and ACTN4 siRNA-treated cells. **(B)** The relative amounts of phosphorylated MRLC to total MRLC in siRNA-treated cells were compared by measuring band intensity. Data represent the mean ± SD of three independent experiments. **(C)** Representative blots showing RhoA pull-down by Rhotekin RBD in control, ACTN1, and ACTN4 siRNA-treated cells. (**D**) Relative bound (active) RhoA protein levels to total RhoA were quantified using the blot shown in C. Data represent the mean ± SD of three independent experiments. n.s., not significant, **P < 0.01.
(TIF)

S2 Fig Transfection efficiency of SW480 cells. **(A)** SW480 cells were transiently transfected with GFP- or ACTN1-GFP-expressing vectors and, after 48 h, imaged by confocal fluorescence microscopy. The merged images of GFP fluorescence and differential interference contrast (DIC) microscopy are shown. Scale bar = 200 μm. **(B)** The transfection efficiencies of GFP or ACTN1-GFP expression were determined as the ratio of GFP-positive cells to total cells at 48 h post-transfection. The results represent the mean ± SD of three independent experiments. n.s., not significant.
(TIF)

S1 Movie Focal adhesion dynamics of a control mCherry-expressing cell. Representative movie of GFP-VCL fluorescence of an mCherry-expressing DLD-1 cell. Live imaging was performed by TIRF microscopy at a frame rate of 1 min/frame. Time is indicated in h:min.
(AVI)

S2 Movie Focal adhesion dynamics of an ACTN1-mCherry-expressing cell. Representative movie of GFP-VCL fluorescence of an ACTN1-mCherry-expressing DLD-1 cell. Time is indicated in h:min.
(AVI)

S3 Movie Focal adhesion dynamics of an ACTN4-mCherry-expressing cell. Representative movie of GFP-VCL fluorescence of an ACTN4-mCherry-expressing DLD-1 cell. Time is indicated in h:min.
(AVI)
