# Supplementary figures and images for: α-Actinin-4 Enhances Colorectal Cancer Cell Invasion by Suppressing Focal Adhesion Maturation
Source: PLoS One. 2015 Apr 10;10(4):e0120616. doi: 10.1371/journal.pone.0120616 (PMC4393021; doi:10.1371/journal.pone.0120616)

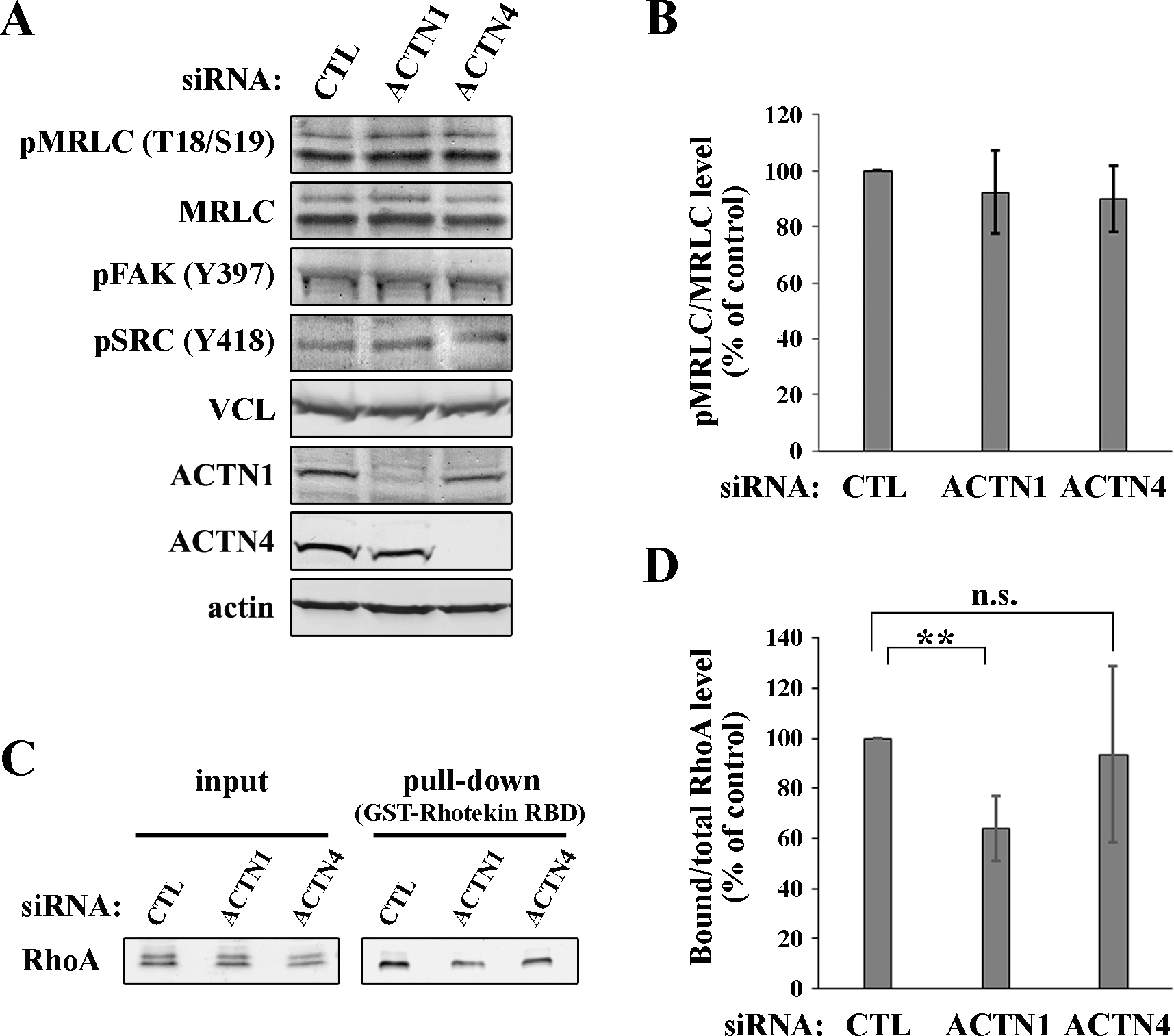

Supplement: S1 Fig — (A) Western blotting of myosin regulatory light chains (MRLC), phosphorylated MRLC (pMRLC), phosphorylated focal adhesion kinase (pFAK), phosphorylated SRC (pSRC), VCL, ACTN1, ACTN4 and actin in control, ACTN1, and ACTN4 siRNA-treated cells. (B) The relative amounts of phosphorylated MRLC to total MRLC in siRNA-treated cells were compared by measuring band intensity. Data represent the mean ± SD of three independent experiments. (C) Representative blots showing RhoA pull-down by Rhotekin RBD in control, ACTN1, and ACTN4 siRNA-treated cells. (D) Relative bound (active) RhoA protein levels to total RhoA were quantified using the blot shown in C. Data represent the mean ± SD of three independent experiments. n.s., not significant, **P < 0.01. (TIF) [file pone.0120616.s001.tif]

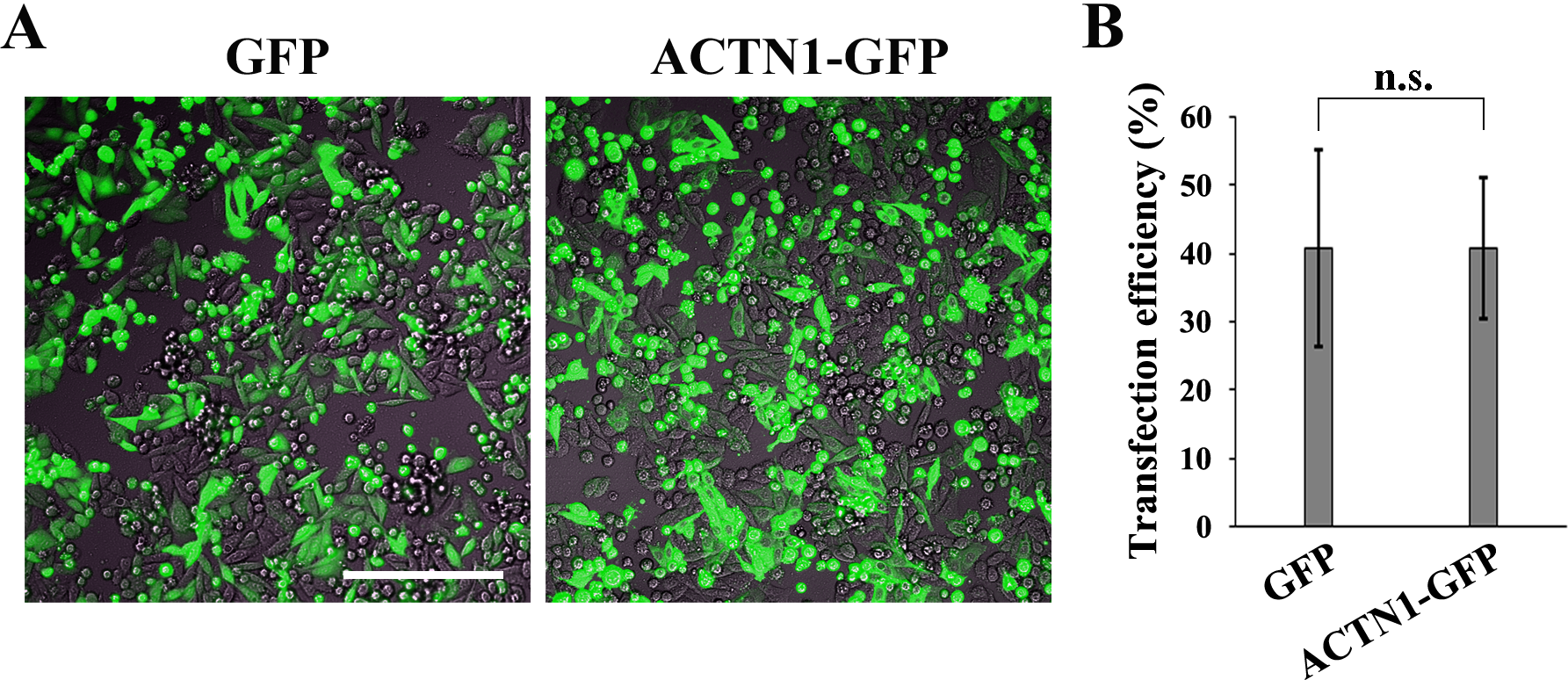

Supplement: S2 Fig — (A) SW480 cells were transiently transfected with GFP- or ACTN1-GFP-expressing vectors and, after 48 h, imaged by confocal fluorescence microscopy. The merged images of GFP fluorescence and differential interference contrast (DIC) microscopy are shown. Scale bar = 200 μm. (B) The transfection efficiencies of GFP or ACTN1-GFP expression were determined as the ratio of GFP-positive cells to total cells at 48 h post-transfection. The results represent the mean ± SD of three independent experiments. n.s., not significant. (TIF) [file pone.0120616.s002.tif]
